# No knowledge gap in human physiology after remote teaching for second year medical students throughout the Covid-19 pandemic

**DOI:** 10.1186/s12909-023-04959-x

**Published:** 2023-12-19

**Authors:** Stefan K. Schauber, Kåre-Olav Stensløkken

**Affiliations:** 1 Faculty of Medicine, Unit for Health Sciences Education, University of Oslo, Oslo, Norway; 2 Centre for Educational Measurement (CEMO), Faculty of Education, University of Oslo, Oslo, Norway; 3 Section of Physiology, Department of Molecular Medicine, Institute of Basic Medical Science, Faculty of Medicine, University of Oslo, Oslo, Norway

**Keywords:** Medical education, Covid-19 pandemic, Remote assessment, Basic sciences, Physiology, Teaching, Knowledge

## Abstract

The COVID-19 pandemic had a disruptive effect on higher education. A critical question is whether these changes affected students’ learning outcomes. Knowledge gaps have consequences for future learning and may—in health professionals' education—also pose a threat to patient safety. Current research has shortcomings and does not allow for clear-cut interpretation. Our context is instruction in human physiology in an undergraduate medical program from high stakes end of term examinations. The sequence of imposed measures to slow the COVID-19 pandemic created a natural experiment, allowing for comparisons in performance during in-person versus remote instruction.

In a two-factorial design, mode of instruction (in-person vs. remote) and mode of assessment (in-person vs. remote) were analyzed using both basic (non-parametric statistics, T-tests) and advanced statistical methods (linear mixed-effects model; resampling techniques). Test results from a total of *N* = 1095 s-year medical students were included in the study.

We did not find empirical evidence of knowledge gaps; rather, students received comparable or higher scores during remote teaching. We interpret these findings as empirical evidence that both students and teachers adapted to pandemic disruption in a way that did not lead to knowledge gaps.

We conclude that highly motivated students had no reduction in academic achievement. Moreover, we have developed an accessible digital exam system for secure, fair, and effective assessments which is sufficiently defensible for making pass/fail decisions.

The COVID-19 pandemic had a global disruptive effect. It fundamentally changed how we taught students, and it changed how students learned. This seemed to be the univocal conclusion after more than two years of social distancing and governmental measures [12, 19, 33]. Obviously, both instruction and assessment had to adapt to the ‘new normal’ of quarantines, as well as to local and national lockdowns. Within a few days, this situation required faculty to develop and use digital platforms to an unprecedented extent. Students were confined in their homes, which drastically changed their teaching and learning environment. A critical question is whether these broad changes affected students’ learning outcomes and performances. If so, it would be worrying if the abrupt changes in teaching, learning, instruction, and assessment led to a deterioration in educational standards [16]. Concerns about eroding academic benchmarks have been expressed, for instance in the UK [2]. Investigation of students’ proficiency levels is especially important in medical education where consequences of knowledge gaps—especially in basic sciences—would hinder students’ future learning [23], diagnostic reasoning [35], and, ultimately, pose a threat to patient safety [15, 24, 31].

Soon after the first wave of COVID-19, several studies addressed possible effects of social distancing on student’s learning, well-being, and academic performance. The general finding in many of the early studies focusing on medical education was that students, globally, reported that they felt the pandemic had a predominately negative impact on their training [4, 7, 14, 17, 26, 28, 30, 36, 37]. Indeed, studies also reported that university students, in many countries, experienced a negative impact of the pandemic especially in relation to their mental health [3, 13, 34]. While this finding is important in itself, there is also ample evidence for the pivotal role of students’ mental well-being, self-regulation, and other psychological factors for learning and academic success [25, 29, 32]. Hence, there is reason to assume that the pandemic affected learning negatively, which in turn might have led to knowledge and attainment gaps.

While students’ negative perceptions of their own learning are a reason for concern, some studies report that actual academic performance was equal to, or even better than prepandemic achievements [1, 26]. For instance, a study using data from a prescription-skills exam in the UK found no differences in performance on a remote (online) exam as compared to in in-person offline administration [18]. Furthermore, a study by Jones et al. [20] found evidence for increased pass rates in online-proctored professional credentialing exams. At the same time, the authors discussed the score-comparability between the different forms of assessment. Such issues in comparability might be critical for the interpretation of results from standardized high-stakes exams which could not be administered in the traditional manner and were changed to other formats, postponed, or even cancelled [10, 22]. Combined, there is reason to assume that knowledge gaps in medical students might be an issue thus far not addressed adequately.

Importantly, there are a number of shortcomings in the research published so far. First, to our knowledge, there is no direct comparison on identical test content in high-stakes contexts with regard to academic achievement. Second, a main obstacle is that effects of the shift in the exam regimen typically are confounded with effects of the shift in the educational environment and instructional approaches. Finally, many studies base their conclusions on data derived from low-stakes assessments or formative tests, where comparability is limited. Combined, these gaps highlight a need for clear evidence for or against knowledge gaps, especially in the context of high-stakes examinations.

Our main research question was whether the pandemic had a disruptive effect on student’s academic achievement that is indicative of knowledge gaps. Our study is set in the context of instruction in human physiology in an undergraduate medical programme. With the beginning of the pandemic, faculty at UiO adopted emergency remote teaching, that is, all courses were re-organized and conducted using digital platforms. Critically, because of the distinctive sequence of the nationally-imposed measures to slow the COVID-19 pandemic, we can analyze data from a natural experiment in which students were allocated to a two-factorial design, the first being mode of instruction (in-person vs. remote) and the second being mode of assessment (again, in-person vs. remote). Hence, we overcome many of the limitations of earlier studies and contribute to a better understanding of the consequences the pandemic-related shifts in assessment and instruction had for students’ academic performance.

## Methods

### The COVID-19 pandemic and its effect on teaching and instruction in Norway

When the pandemic struck Norway, the government practically shut down society from the 13th of March 2020. In the first wave of the pandemic, teaching at the University of Oslo was delivered digitally and off-campus. On-campus teaching was possible under certain restrictions (i.e., distancing measures) and for specific courses, including elective courses in anatomy and small group teaching in clinical settings. For human physiology, teaching was digital and off-campus from March 2020 to February 2022. Between the first and the second COVID waves, the end of term exam, autumn 2020, was held on-campus in a testing center where cohorts of up to 130 students sat one exam simultaneously (like an in-personpre-pandemic exam). Since April 2022, only in-person teaching has been provided.

### Educational context

The medical programme at the Faculty of Medicine at the University of Oslo is divided into pre-clinical and clinical parts. The first two years cover basic sciences, with emphasis on cell biology, biochemistry, and statistics in the first year. In the second year, the curriculum focusses on physiology, anatomy and propaedeutics. In the third year, students enter clinical education, which lasts until they finish their undergraduate education in the sixth year. In human physiology, the focus of this study, the courses last for 38 weeks before ending with a high-stakes exam. Both pre- and post-pandemic in-person teaching consists of traditional lectures for all students in auditoriums (eight hours per week), where providing recordings of lectures is voluntary for the teacher (about 50% record their lecture on video). Attendance is voluntary for students. In addition to traditional lectures, student active learning covers twelve hours of instruction per week and includes courses, small-group teaching, and team-based learning. Finally, five hours per week is reserved for self-study.

Student active learning in physiology includes interactive courses (spirometry, respiration physiology, exercise physiology, etc.), where students perform basic experiments in groups and discuss questions related to the assignment with each other and the teacher. Small group teaching is 12 students per teacher, two hours per session, 13 sessions per semester. Students in each session must be prepared to work on a given assignment. These two teaching formats are mandatory for medical students in Oslo. Team-based learning (5 sessions each semester) is voluntary and offered according to a standard format, including individual readiness assurance tests, team readiness assurance tests, clarification session, application exercises and peer evaluation.

Since students were confined to their homes during the Covid-19 pandemic, the normally in-person lectures were delivered online. Teachers reported low student activity during lectures and few questions were asked. It was debated whether students should be required to turn their cameras on, but this was not enforced for privacy considerations. At the same time, teachers were obliged to record the lectures. Small group teaching was performed in the same manner, where each teacher opened a digital room using a video conferencing platform and had discussions with the students (again, these were mandatory for students). Finally, attempts were made to provide digital interactive courses, but with no or very reduced possibility to assign practical exercises. However, breakout rooms for discussion were frequently used. In summary, although emergency remote teaching was done fully remotely and digitally, students received approximately the same number of hours of instruction as before the pandemic.

### Study design

**Fig. 1:**
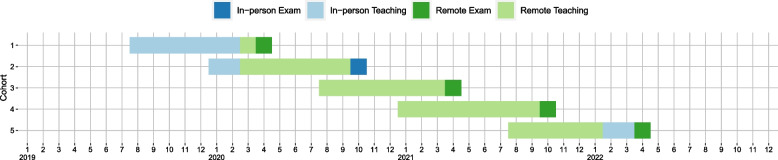
Timeline for implementation of different modes of teaching and exams

The medical programme in Oslo has two intakes per academic year with approximately *N* = 125 students per term. Hence, exams are given both in spring and autumn (denoted “-1” and “-2”; e.g., 2019–1 and 2019–2 refer to the spring and autumn exams in 2019, respectively). Measures enacted to slow the COVID-19 pandemic provided a unique opportunity to test students’ academic performance during the pandemic from the perspective of a natural experiment. The shifts in type of instruction and mode of assessment during the pandemic are given, chronologically, in Fig. 1. Table 1 summarizes the two-factorial design given by the natural experiment and details cohorts included in this study. From the pre-pandemic exams, we included all available data beginning with 2017–2 since these were stored in a format that allowed easy digital processing. Most importantly, this design allows us to disentangle the effect of mode of instruction and the effect of type of assessment.

**Table 1:** Overview of the different student cohorts and if they were exposed to in-person or remote teaching, in-person or remote exam

		Teaching	
		In-person	Remote
Exams	In-person	2017–2, 2018–1, 2018–2, 2019–1, 2019–2	2020–2
		*N* = 526	*N* = 106
	Remote	2020–1	2021–1, 2021–2, 2022–1
		*N* = 116	*N* = 347

### In-person exams: Pre-pandemic procedure

#### Test construction procedure

The development of test content is usually the first step in assembling an assessment. Teachers involved in the relevant module prepare exam questions from their own instruction. The exam committee, consisting of five faculty members, then reviews these questions to ensure appropriate content and that questions cover the module’s learning objectives.The selected-response questions used in the exam need to align with general rules for developing exam questions, which are adapted from the National Board of Medical Examiner’s item writing guides [8, 9].

The specifically developed questions are then distributed according to a fixed blueprint based on the curriculum, resulting in approximately 125 questions in the exam. A major objective for the exam committee is that the exam questions trigger reasoning and only to a lesser extent mere recall of factual knowledge. In general, four types of response-formats are used:1) items in single-best answer format, 2) multiple response items (Pick-N), 3) extended matching (‘pull-down’ items) and 4) short essays where students reply in free text with typically up to four sentences.

#### Pre-exam quality assurance

The exam committee applies several procedures to ensure sufficient item quality. Senior medical students, one external assessor, and two external faculty members read and comment on the exam. The exam committee reviews the suggestions and revises the questions in joint meetings. The questions are then sent to the administrative team, checked for spelling mistakes, and put into the exam system (Questionmark Onpremise). Finally, the exam committee conducts one concluding round of proofreading and performs a mock exam using the same software as the students.

#### Exam administration

A whole cohort of students simultaneously sits the test in one computer lab. Due to local regulations, an in-person exam lasts for up to five hours; hence, students can use up to 2.4 min to answer a question, on average. The exam software registers the time used per student and, in the period between 2016 and 2020, the average time used per question was 1.8 min.

#### Post-exam quality assurance

After the exam and the initial grading of the short essays, but before results are published, the Centre for Health Sciences Education provides a psychometric report to the exam committee. This report serves as feedback to the exam committee and forms the basis for discussing quality of the questions in light of students’ performance. The report visualizes the main properties of the exam including, for each question, quantitative information such as item difficulty (i.e., item facility, percentage correct answers), item discrimination (i.e., the correlation between item-performance and total score) as well as a graphical distractor analysis. The exam committee evaluates the exam holistically, that is, considering the psychometric report, their own expertise, and students’ comments on the exam. For instance, the committee discusses difficult questions specifically and can decide to exclude items from the exam if they are flawed, incorrectly formulated, too specific or misleading. After the meeting, the exam results are published, together with a report of excluded questions. The students receive grades on an A (excellent) to F (fail) scale. Students also receive an email with detailed feedback on their relative performance in different subject areas (i.e., a grade in each subject). Finally, about 20% of the exam questions are published after the exam. The remaining 80% are used to further improve the item database and to create new exams and re-sit exams.

### Remote exams: video-based oral exams during the COVID-19 pandemic

As in-person exams could not be administered due to pandemic restrictions, assessments had to be adapted. The Faculty of Medicine gave the exam committees freedom to develop and evaluate exam systems fit for the purpose. Importantly, the grading (A-F) was abandoned and only pass/fail was used. The adapted exams had to be approved by the dean of education. In human physiology, the exam committee decided to conduct a video-based oral exam that uses previously developed, quality-controlled, but unpublished material from the pre-pandemic in-person exams. A major aim was to ensure that all students received questions which covered the curriculum adequately and were evaluated to a uniform benchmark. For each exam administration five item sets were provided (four used, randomly picked and the fifth set served as back-up) and each item set consisted of unique 15 questions each, using a downsized blueprint of the original exam. Each set was only used for half a day; the same set was used for all students during that time.

#### Test construction procedure

For the remote exam, only items were included that: a) were deemed well written, b) did sufficiently discriminate between low-performing and high-performing students, and c) where distractors were well-functioning. For the first two remote exams (2020–1 and 2021–1) the questions were selected from the database of unpublished items in three categories, easy (above 80% correct), medium (between 60 and 80% correct) and hard (between 40 and 60% correct). One set of questions then consisted of 15 multiple choice items, of which 6–7 were easy, 6–8 were of medium difficulty, and 1–2 were hard. Finally, the exam committee ensured that the selected questions covered the curricular learning objectives adequately. For the two latest remote exams (2021–2 and 2022–1) the procedure was adapted and the exact item difficulties (i.e., percentage correct per item) from previous exams were used to calculate the average difficulty level of each item set.

#### Pre-exam quality assurance

Item sets were piloted to assure comparable time demands. Students were informed about the details of the remote examination in a special lecture 30 days before the exam. Each exam was conducted by two examiners—one internal and one external. The internal examiners were selected from the pool of faculty teachers in the corresponding course and from the members of the exam committee. The external examiners were recruited from a list provided by the faculty administration. The faculty administration organized pairs of examiners and randomly distributed examinees to these teams. The students were notified one week in advance of their examination day.

#### Exam administration

Two versions of each item set were provided in Microsoft Word to the examiners. One version was to be shown to the students taking the exam. The second version provided the correct answers as well as a guide with additional background on the learning objectives to make sure that all examiners had sufficient information about the subject of each question. Both versions were sent to all the examiners a few days before the exam.The exam committee provided a two-page written instruction on how to conduct the exam, which was also sent to the examiners in advance. A joint videoconference with all the assessors was held two days before the exam, to ensure a similar procedure in all teams.

The exam was administered over two days, with one additional day in reserve if technical problems should arise during the exam (not used in any of the exams). The local IT department was on standby, with a designated video room for technical assistance. Each pair of examiners conducted four examinations before lunch and four examinations after (i.e., they evaluated eight students per day). Prior to the exam, the assessor teams received the examinees’ e-mail addresses and phone numbers from the administrative office. Students received their time slot (before and after lunch) one day prior to the actual exam. Importantly, students had to be available for the exam for the entire time period; no exact time slots or order of the candidates were announced prior to the exam, to minimize the possibility of cheating. On the exam day, the examiner phoned the examinee and asked her or him to log onto the video-conferencing tool. After an ID-check and securing that the examinee’s room was empty by sweeping with the webcam, the student was informed about the practical procedure of the exam. No headphones or earplugs were allowed.

The assessor team shared the Word document and started the examination when the student confirmed that she or he could read the questions. The student then had 20 min to respond to the 15 questions.. The examiners noted the time used and reported that students used their 20 min, using an average of 1.3 min per question. If the student received a score of 65% or higher, the exam was passed and finished. A student who scored below 65% was given three additional short-essay questions to support a pass/fail decision. These questions were also shown on the screen, but the scoring rubric for points needed to pass was visible only to the examiner. This guide typically consisted of key responses in the form of bullet points that the student had to mention in reply to the question. It was clearly stated in the guide that if these key responses were not included, the questions were failed. Two of the three short-essay questions had to be answered satisfactorily for the student to pass. Typically, these three questions focused on learning objectives from the central part of the curriculum which were not covered in the 15 other items. The combination of selected response and short essay questions allowed for a rapid screening of passing students, and careful evaluation when the commission were unsure of pass/fail. The behavior of the students was on average pleasant but concentrated. The students clearly read each question, reasoned and reported an answer. The short time and observation of eye movement makes us quite sure no other helping notes were used.

#### Post-exam quality assurance

Because the exam was administered as a remote exam with oral response, no data about the students’ responses to individual items were recorded systematically. Consequently, no post-exam psychometric analysis was possible. However, the overall exam results per student were recorded. Hence, average scores on the administered item sets could be compared to the average scores on equal item sets before the pandemic. Out of the five item sets developed for the 2020–1 exam, three were reused for 2021–1. An ad-hoc analysis of students' responses to these exam sets implied that the selection of questions had been comparably easy. In turn, the exam committee decided to assemble new item-sets for the following administrations. These new sets were still based on the item statistics available from previous exams, but the process was adjusted to include questions that were better aligned with the in-person exams’ level of difficulty.

### Analytic approach and statistical inference

While our study design is similar to a two-factorial design, we could not compare performances across all modalities using one single statistical approach. This is due to the distinct types of data sources. Hence, to address our research question, we carried out three comparisons: First, we contrasted performances using identical item-sets that were administered either in the remote exams or in in-person exams. Second, we compared exam scores from both remote and in-person exams using a resampling approach. Finally, we compared scores following either in-person teaching or remote teaching using a linear mixed effects model.

The first comparison was carried out descriptively and we inspected differences graphically. In order to draw inferences on differences in performances on identical item-sets, we used a Student T test (conducted in Graph Pad Prism; vs 9.3.1) and a binomial test (conducted in R).

#### Resampling: Using subsampling to compare performances across modalities statistically

Our study investigates differences in performances before and during the pandemic. Hence, we aim to compare the average scores observed on the remote exams to their equivalent on the in-person exams before the pandemic. To make these two types of exams comparable, and to arrive at statistical conclusions, we applied a resampling procedure. Out of the five pre-pandemic in-person exams (2017–1 to 2019–2; *N* = 617 items and *N* = 526 students, i.e., *N* = 64,897 observations in total) we sampled 10.000 datasets, each consisting of 30 students and 15 items. We refer to these datasets as “synthetic” exams since we were using the same rationale as for the remote exams when sampling the datasets. This approach enabled us to calculate the mean and its confidence limits for scores on pre-pandemic exams that then are comparable to the remote exams during the pandemic.

We included items that either had a discrimination of r_(is, ts)_ >  = 0.25 or higher (i.e., the correlation of item-score with the test-score on the full exam) or had been answered correctly by at least 90% of the students. Then, for every synthetic exam we first selected, randomly, five easy, eight medium, and two difficult questions. This corresponded to the actual distribution of items across the three broader categories in the first two sets of remote exams (2020–1, 2021–1). In a second step, we randomly selected *N* = 30 students that had answered these 15 questions in the corresponding in-person exam. We then calculated the students’ performances on these 15 questions based on their actual answers. We repeated this procedure 10,000 times.^1^

For the resulting 10.000 synthetic exams, we calculated the arithmetic mean and its 95% confidence limits. We performed a two-tailed test, meaning that we determined statistical significance by cutting the lower end of the distribution of the resampling-distribution at 2.5% and the upper tail of the distribution at 97.5%. Values that fell either below or above the corresponding threshold were considered statistically significant at the 5% level. The subsampling procedure was implemented in the R Language and Environment for Statistical Computing [27].

#### Linear mixed-effects model

For the in-person exams administered either during in-person or remote teaching, both person- and item-level data were available. Hence, we use a linear mixed-effects model to examine differences in performance and include the scores per question as the dependent variable while a factor for remote or in-person teaching was entered as the independent variable. We include random effects for students and items and a fixed effect for in-person teaching vs. remote teaching. We used the R packages lme4 [6] and sjPlot [21] to estimate the model, to determine statistical significance, and to produce the resulting tables.

#### Psychometric analyses

In terms of psychometric properties, we estimate reliability by calculating Cronbach’s Alpha for both the in-person exams and the synthetic exams (i.e., the randomly drawn datasets resembling the remote exams). Following the literature, we regard an Alpha coefficient of 0.8 or higher as appropriate for high stakes examinations [11]. For the synthetic exams, we also calculate the correlation between students’ performance on the remote exam and the performance on the in-person exam. This allows us to evaluate the degree to which a 15-item subset from the synthetic exam was indicative of the performance on the full, in-person exam. For the actual remote exams, no item-level data was available. Hence, it was not possible to determine reliability coefficients for that given subset.

Reliability estimates are in general dependent on test length (number of items) and the standard deviation of the observed scores (between-person variance). Since, for the synthetic exams, the number of items is constant, we correlated the reliability estimates with the standard deviation in the specific synthetic exams. If that correlation is high, it indicates that variation in reliability is likely a result of the specific student scores sampled being homogenous. Put differently, low reliability is due to low score-variation.

## Results

### Descriptive statistics

In total, we include results from *N* = 1095 students who took either in-person or remote exams between 2017–2 and 2022–2. There were *N* = 463 students who took the remote exam during the COVID-19 pandemic. For the in-person exams, both before and during the pandemic, there were *N* = 632 students. In general, no missing data was observed.

### Exam composition

**Fig. 2:**
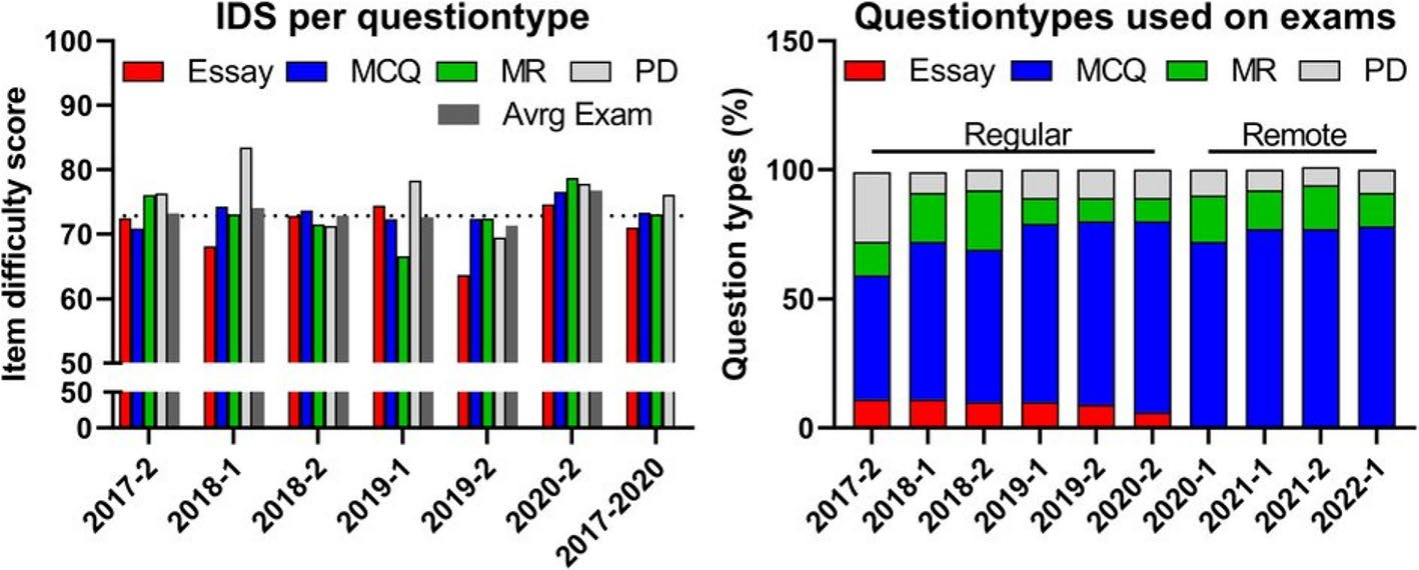
**A** Historical data on item difficulties on in-person exams during in-person teaching, in-person exams (2017–2 to 2019–2) and remote teaching, in-person exams (2020–2). **B** The relative share of questions types used on exams from 2017–2 to 2022–1 (‘Regular’ = in-person exams, *MCQ* Single best option, *MR* Multiple response, *PD* Pulldown/extended matching)

The number of items per in-person exam varied slightly across administrations, with 121 items in the exam for 2018–2 (minimum), and 125 items for 2019–2 (maximum). Figure 2A shows average item difficulty per response format during the period between 2017 and 2019 (72.8 ± 1.0) as well as for the in-person exam after remote teaching (2020–2; 76.8). In addition, Fig. 2A also indicates some variations in item difficulty between the utilized question types. The relative share of the different response-formats for both the in-person and the remote exam is given in Fig. 2B. The share of single-best answer multiple choice questions was generally highest, ranging from 48% in 2017–2 up to 74% in 2020–2. For the remote exams, the first 15 questions in general were selected-response items. However, for borderline candidates, three essay questions were used to differentiate between failed and passed (see paragraph “Exam administration”).

### Psychometric properties

Reliability estimates for the in-person exams were generally higher than Cronbach’s Alpha = 0.80. Across the in-person exams, estimates ranged between α = 0.83 for 2017–2 and α = 0.88 for 2018–1. The in-person exam during remote teaching in autumn 2020 had a comparable reliability estimate with α = 0.83. For the 10.000 synthetic exams (15 items, 30 students) reliability estimates were, on average, lower with a median Alpha of α = 0.58. Across all synthetic exams, the alpha coefficient correlated strongly with the standard deviation of students’ scores (*r* = 0.93). Since the number of items for these subsets is constant, this correlation indicates that the low reliability estimates for the synthetic exams were mainly attributable to low between-person variation in the subgroup drawn. Importantly, in addition we correlated the students’ scores on the full in-person exam with their respective scores on the subset of 15 items in the synthetic exam. The average correlation across the randomly selected short exams was Spearman’s rho = 0.78, indicating a substantial overlap between students’ rank orders in the shortened synthetic exams and the full in-person exams.

### Overall results from the remote exam

**Fig. 3:**
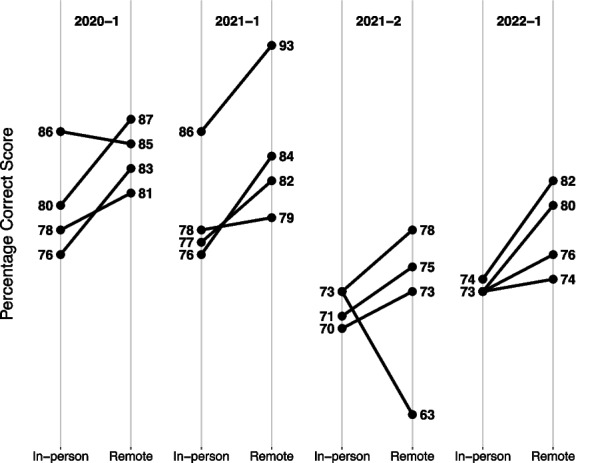
Students’ scores on equivalent sets of items across the four remote exams held between 2020 and 2022 in Spring («-1») and Autumn («-2»). Scores on the comparable in-person exams were obtained in the pre-pandemic period between 2017–2 and 2019–2

**Fig. 4:**
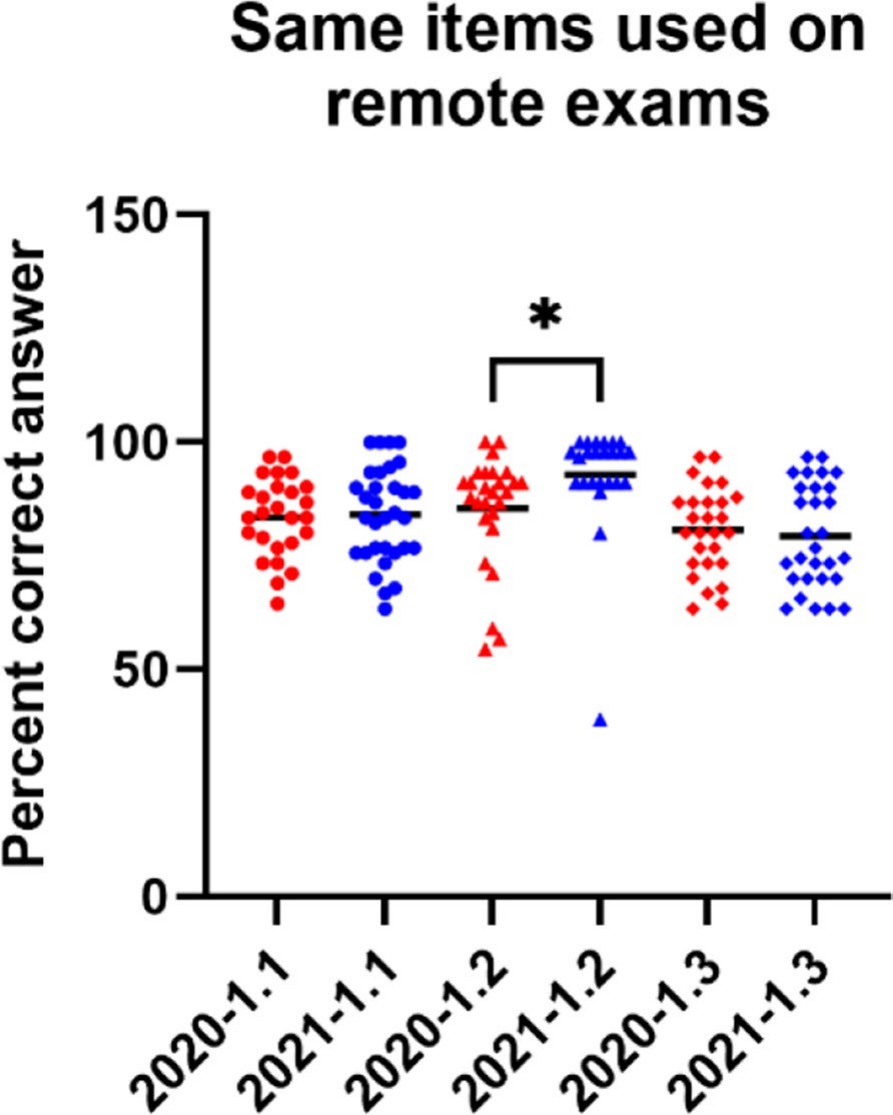
Three sets of 15 questions were used twice; 2020–1 and 2021–2. In one of the item sets, the students performed better after remote teaching (*p* < 0.05, student t-test)

Students on the remote exam received higher scores on average compared to the equivalent item sets used in the pre-pandemic in-person exams. Detailed scores for all administrations are given in Fig. 3. The general tendency that students scored higher on the remote exams than on equivalent sets of items in an in-person exam was confirmed by a binomial test. For the 16 item sets that were used in both the in-person and remote exams, students performed better on 14 and worse on 2. A binomial test indicated that this pattern was statistically significant (87.5% observed vs. 50% expected as null hypothesis; *p* = 0.004). In addition, for the three item sets that were used in two remote exams (2020–1 and 2021–1), a Student T-test showed that the students performed better in one out of three item sets (*p* = 0.04, df = 49, t = 2.126; Fig. 4). The differences for the other two item sets were not statistically significant.

### Subsampling procedure: Statistical comparison of scores on remote exams to pre-pandemic performances

In the subsampling procedure, the average percentage correct score for a synthetic exam (15 items, 30 students) was 67.9%. The 95% confidence limits ranged from 61.2% to 74.5% correct score. As a plausibility check, for each of the 10.000 synthetic exams we calculated the difference between the original item difficulty per item category (i.e., for the easy, medium and difficult items) in the item sets used for the remote exams and their difficulty in the synthetic exam. The average difficulty in the three item categories was 55%, 72.3%, and 88.9% in the remote exams and 54.2%, 71.3%, and 88.2% in the synthetic exams.

### Performances following in-person teaching: Comparing scores on in-person exams with scores on remote exams

**Fig. 5:**
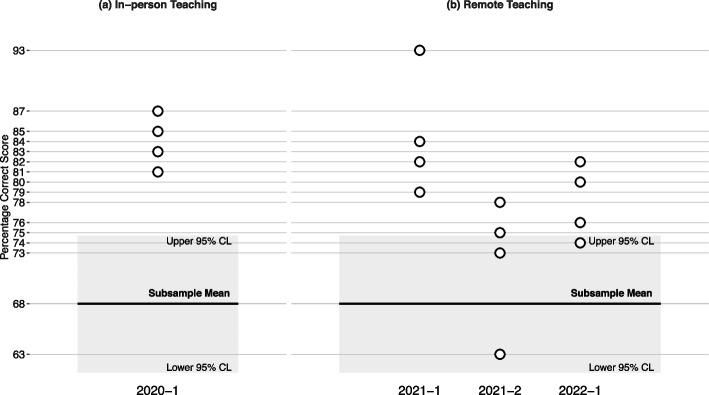
Students’ academic performance on remote exams after either in-person teaching (**a**) or remote teaching (**b**) compared to in-person exams based on a subsampling procedure. *Note*. *CL *Confidence Limit. The x-axis shows the exam term, beginning with 2020–1. The y-axis shows percentage correct scores. The dots show performances on the four itemsets from the remote exams. These can be compared to the bold horizontal line (subsample mean), which corresponds to the average percentage correct score across the 10.000 synthetic exams. The grey area signifies the 95% confidence limits derived from the resampling procedure (Lower 95% CL and Upper 96% CL). The lower and the upper edge of the grey area cut 2.5% on the lower end and upper end of the resampling distribution, respectively

Average scores on the four item sets used in the first remote exam (2020–1) ranged between 81 and 87% correct. Figure 5-a presents the direct comparisons of the average scores on these item sets following in-person teaching in the 2019/2020 winter-term. When contrasted to the confidence limits [61.2%, 74.5%] derived from the subsampling procedure, the results indicate that scores in these four item subsets were significantly higher than what would be expected from performances in equivalent subsets of items on in-person exams. Thus, we do not find evidence for lower scores on the remote exam following in-person teaching.

### Scores on the remote exams following remote teaching compared to scores on in-person exams following in-person teaching

Three cohorts of students took remote exams following remote teaching. In total, twelve item sets were used. Results from these exams are given in Fig. 5-b and show that scores on nine out of the twelve item sets exceeded the upper 95%-confidence limit. Three scores fell within the confidence limit and none below. Hence, in contrast to our expectation, we did not find evidence of knowledge gaps after remote teaching.

### Scores on regular exams following either in-person teaching or remote teaching

Furthermore, we compared students’ performance on the pre-pandemic in-person exams to the in-person exam in autumn 2020 (2020–1) using a linear mixed effects model. We included random effects for students and items and a fixed effect for whether the exam was held after in-person or remote teaching. The main effect for exam performance on the in-person exam during remote teaching was not statistically significant, with p = 5.1%. The comparable standardized beta coefficient was positive, but weak (β_Std_ = 0.04; CI 0.00–0.07; for details see Table 2).

**Table 2:** Results from mixed effects models comparing students’ scores on in-person exams following either in-person teaching or remote teaching

	**Null Model**			**In-person/Remote Comparison**		
**Fixed effects**	Estimate [CI]	std. Beta [CI]	*p*	Estimate [CI]	std. Beta [CI]	*p*
(Intercept)	4.41 [4.32; 4.50]	0.00 [-0.04; 0.04]	< 0.001	4.37 [4.27; 4.46]	0.00 [-0.04; 0.04]	< 0.001
Remote teaching				0.24 [-0.00; 0.48]	0.04 [-0.00; 0.07]	0.050
**Random Effects**						
Item variance	1.22 _item_			1.21 _item_		
Student variance	0.22 _stud_			0.22 _stud_		
Residual variance	4.24			4.24		
Total observations	77,723			77,723		

### Comparison of fail rates across exams

To validate the results, we inspected pass-rates and the number of students who withdrew from the exams. We compared both fail rates and non-attendance of eligible students for the exams held in the pandemic to pre-pandemic numbers from 2017–2 to 2019–2. The exam with the highest rate of withdrawal and lowest fail-rate was the 2020–1 remote exam. Both the in-person exam in 2020–2 and throughout 2021/2022 had comparable rates of withdrawals and fails compared to the pre-pandemic exams. None of the Chi-squared tests indicated a statistically significant higher rate of withdrawals from the exam, as compared to the pre-pandemic average. The only statistically significant difference was found for the fail rates in the 2020–1 exam, where the fail rate was lower than in the period from 2017–2 to 2019–2. Detailed results are given in Table 3.

**Table 3:** Number of eligible students who either did not take part in the exam (no-show/sick) or who failed the exam. Chi-squared tests were carried out to compare the numbers between the pre-pandemic and the pandemic exam administrations

Cohort	Teaching	Exam	Eligible	No-show or sick	Failed
2017–2 to 2019–2	In-person	In-person	*N* = 526	*N* = 29	*N* = 32
2020–1	In-person	Remote	*N* = 116	*N* = 9 (*p* = 0.50; χ^2^ = 0.45; df = 1)	*N* ≤ 6 *p* ≤ .05^a^
2020–2	Remote	In-person	*N* = 106	N = 8 (*p* = 0.58; χ^2^ = 0.30; df = 1)	*N* ≤ 6 *p* > .05^a^
2021–1 to 2022–2	Remote	Remote	*N* = 347	N = 14 (*p* = 0.44; χ^2^ = 0.58; df = 1)	*N* = 16 (*p* = .47; χ^2^ = 0.51; df = 1)

^a^Since individuals could be identifiable in very small groupings, we do not give the exact statistics and numbers here

## Discussion

The COVID-19 pandemic has changed learning, instruction, and assessment globally. Against this background, we investigate whether there is evidence indicative of knowledge gaps. Waves of national lockdowns created a two-factorial designed natural experiment, allowing us to analyze the effect of remote versus in-person teaching and remote versus in-person exams in end-of term examinations in human physiology taken by second-year medical students. The main findings were that students received higher scores on item sets in the remote exams when compared to equal item sets in previous in-person exams. When we compare performances on an in-person exam conducted in a period with less restriction on social distancing, but with maintained remote teaching, we find a similar tendency. We interpret these findings as empirical evidence that both students and teachers adapted to the pandemic disruption in a way that did not lead to detectable knowledge gaps, at least in the specific subject (human medical physiology) studied here and in highly motivated medical students.

Our results are in line with previous findings within health professions education that did not indicate evidence of knowledge gaps in relation to the Covid 19 pandemic, and we add evidence to this body of research. More specifically, we provide data that compares the in-person and remote administration of teaching and testing before and during the covid-19 pandemic. Such comparisons are rather rare in the current literature. While we do not find evidence of knowledge gaps, there are also several key differences from similar research. Importantly, as Hamamoto Filho et al. [16] discuss, consequences due to a lack of in-person teaching might be more severe in low-and middle-income countries. Indeed, the authors provide evidence for a lack of development of knowledge, using longitudinal data from progress tests. This points to a wider issue, namely that most studies so far use cross-sectional designs. Hence, there is only a limited ability to follow and analyze students’ learning trajectories across the course of study. One further difference of our approach to other studies is that we could not directly include measures of students’ mental health [1, 26]. Therefore, we were not able to relate students’ perceptions of their learning and mental health to their academic performance.

During the COVID-19 pandemic, universities had to adapt to the new normal of remote teaching and faced disparate challenges. Importantly, the literature highlights discrepancies in students’ access to adequate technology [7], a problem that again might be more consequential for low-income students and/or countries. For the ‘Global North’, we assume that the teaching approach at the University of Oslo is comparable to that of other academic institutions, with digital lectures, digital small group teaching and adapted courses with limited student activity. In our context, we speculate that a rapid adaptation to the pandemic disruption was facilitated by the availability of such resources for both teachers and students. Furthermore, medical students are typically highly motivated and self-driven, which is, most likely, the case in other medical schools, too. This, to a certain extent, might make them more independent of the actual instructional environment [13, 29]. Taken together, these observations could point to a wider application of our results and indicate a minimal impact of the pandemic on student academic achievements.

While these results are encouraging, we caution against interpreting our findings as evidence for students performing *better* due to digital remote teaching. While our statistical analysis supports this observation, our study was not designed to draw a conclusion on the actual nature of increased performance. Obviously, other factors such as increased time spent studying outside of class or assessor leniency could have affected the results presented here. Further, a difference between remote and in-person exams was the number of questions. In the remote exam, students had to focus for 20 min, with 1.3 min per question, while an in-person exam would last for five hours with 2.8 min per question. Differences in performance might be attributable to less mental strain and less cognitive exhaustion when taking the shorter exams. Importantly, however, the resampling approach indicates that none of the performances were significantly below what could be expected. Hence, we still find no empirical evidence of erosion of educational standards.

A secondary outcome in our study is that we developed a remote exam system which according to our results tested academic performance in an effective and fair manner. First, no additional exam software is required. Rather, standard software used for videoconferencing and text editing was used. The remote exams covered contents comparably since questions were spread according to the same blueprint as for the original exam, albeit with fewer questions. Overall analysis indicates that, in the given context, those shortened exams were reasonably robust. In addition, although not systematically investigated, students gave positive feedback on the transparency of the approach and the passing criteria for the oral examination. Finally, we provided a high-stakes exam which was, by design, not affected by examiners' stringency or leniency.

An important catalytic effect was that the examiners could directly observe students’ reasoning on a given question and why they opted for a correct answer. Examiners reported that this provided valuable insight into what makes a good exam question. The utilization of short-essay questions allowed for adapting the exam to better differentiate between borderline students and to assign pass/fail grades appropriately. In addition, the fail rates across both remote and in-person exams seem comparable. While the first remote exam in 2020–1 had a significantly lower fail rate, we argue that our data supports the conclusion that fail rates were otherwise relatively stable across administrations. The exam results, especially in the first wave of the pandemic, might have been influenced by examiners’ increased leniency. Specifically, one might speculate whether the selection of items within the first wave was biased in students’ favor.

One main change with the pandemic was that teaching, learning, and assessment became more distributed, both physically and temporally. Our study suggests that robust and effective remote exams are feasible, especially in times of a global crisis. This can be accomplished without the effort and resources needed for conducting safe exams using proprietary remote-proctoring software. An important aspect of the approach utilized in our context was that rich and valid information from prior administered exams was available. Hence, our study underpins the importance of continuous work focusing on fostering and increasing exam quality in order to produce defensible assessments any time, but especially during times of crisis.

One limitation of the approach to remote exams discussed here is that students could share information on the exam content. In that case, results would be compromised. One measure the exam board took was to randomize students to time slots and item-sets, thus making it unlikely that students could share information on the particular questions posed. Based on feedback from the committees in joint meetings after the exam, there was no indication of collaboration or any other cheating behavior. Taken together, a large effort was made to reduce the chance of collaboration between students. An important further limitation is that our study focuses on cognitive aspects of the learning objectives in a physiology class within basic science teaching. One of the concerns raised by students and teachers was that clinical, patient-centered instruction was most affected by the pandemic [17]. While this is obvious, there is a need to stress the importance of knowledge in subjects such as anatomy, biochemistry, or physiology for high-quality patient care. Research highlights the role of sound understanding of basic sciences for students' future learning in the clinical domain, but also for unexpected situations in the clinical context after graduation. Our findings provide some reassurance that both teaching and learning were sufficiently aligned.

In conclusion, our study highlights that, locally, instruction was well adapted to the disruption of the pandemic. Importantly, previous work on quality assurance was instrumental in develop a fair and feasible exam regime. Finally, the approach described here may point to possibilities for new assessment practices that maintain high quality standards and are more flexible and adaptable.

## Data Availability

Access to data will be made available upon reasonable request. In that case, please contact the corresponding author (Stefan K. Schauber).

## Footnotes

1 We conducted a series of robustness checks with different criteria for item selection (i.e., we varied discrimination and difficulty thresholds). None of these affected the outcomes substantially.
